# Ambiguity and Unintended Inferences About Risk Messages for COVID-19

**DOI:** 10.1037/xap0000416

**Published:** 2022-05-19

**Authors:** Dawn Liu Holford, Marie Juanchich, Miroslav Sirota

**Affiliations:** 1 Department of Psychology, University of Essex

**Keywords:** COVID-19, risk perception, public health messaging, risk events, pragmatic inferences

## Abstract

The World Health Organization established that the risk of suffering severe symptoms from coronavirus disease (COVID-19) is higher for some groups, but this does not mean their chances of infection are higher. However, public health messages often highlight the “increased risk” for these groups such that the risk could be interpreted as being about contracting an infection rather than suffering severe symptoms from the illness (as intended). Stressing the risk for vulnerable groups may also prompt inferences that individuals *not* highlighted in the message have lower risk than previously believed. In five studies, we investigated how U.K. residents interpreted such risk messages about COVID-19 (*n* = 396, *n* = 399, *n* = 432, *n* = 474) and a hypothetical new virus (*n* = 454). Participants recognized that the risk was about experiencing severe symptoms, but over half also believed that the risk was about *infection*, and had a corresponding heightened perception that vulnerable people were more likely to be infected. Risk messages that clarified the risk event reduced misinterpretations for a hypothetical new virus, but existing misinterpretations of coronavirus risks were resistant to correction. We discuss the need for greater clarity in public health messaging by distinguishing between the two risk events.

## Public Significance Statement

Five studies demonstrated the ambiguity of “at increased risk” messages. People misinterpreted that individuals at increased risk were more likely to contract a virus, not just to suffer severe symptoms (as the message intends). In addition, people perceived that nonvulnerable individuals were less likely than vulnerable ones to be infected. Risk messaging that clarifies what exactly is at risk could reduce misinterpretations.

Clear communication about risks is essential when faced with a new and severe public health threat such as the coronavirus disease (COVID-19).^1^ Containing the disease requires substantial changes to public behavior—for instance, practicing social distancing and raising hygiene standards (World Health Organization [WHO], 2020b). To appreciate the need to change their behavior, people must understand the likelihood of contracting and developing severe symptoms from COVID-19. Many health authorities have therefore developed and promoted risk information campaigns highlighting the potential consequences of COVID-19. Risk communication in many countries details the consequences of the illness by describing some groups as being “at increased (or higher) risk from coronavirus” (see Table 1). In this article, we studied how people understand what it means for vulnerable individuals to be “at risk from coronavirus,” whether people are confused about what the risk refers to, and whether focusing the messages on a particular vulnerable group leads to unintended inferences about the risks faced by the rest of the population.[Table tbl1]

## Ambiguity and Possible Misinterpretation of What is at Risk for Vulnerable Groups

Risk messages are often ambiguous, meaning that it is unclear what exactly is risky (Gigerenzer & Edwards, 2003; Gigerenzer & Galesic, 2012; Harris & Corner, 2011; Nygren et al., 1996; Slovic & Lichtenstein, 1968). For COVID-19, “at risk” could refer to the probability of contracting the disease (i.e., becoming infected), the probability of suffering severe symptoms from the disease (Bi et al., 2020; Jones et al., 2020; Onder et al., 2020; Verity et al., 2020), or other health consequences, such as the probability of long-term COVID-19 symptoms (Nabavi, 2020). Current evidence suggests that social and behavioral factors (e.g., the motivation and ability to practice social distancing), rather than intrinsic characteristics (e.g., age, gender, or ethnicity), influence people’s likelihood of contracting the disease (e.g., Jones et al., 2020). In contrast, there is strong evidence that certain intrinsic characteristics mean some people are more likely to suffer severe symptoms from COVID-19, such as being over 70 years of age or having underlying medical conditions (Onder et al., 2020). The different probabilities for contracting a disease and for suffering severe symptoms from it are not unique to COVID-19. For example, the probability of getting the flu is about 3%–11% and comparable across age groups (Tokars et al., 2018). However, the probability of hospitalization is higher for adults over 65 years of age than younger adults. In the 2018–2019 U.S. flu season, for example, the national authority estimated that overall, only 1%–2% of flu cases in the population resulted in hospitalization in the overall population, but this rate of hospitalization was 5–10 times greater among over-65s, at 9%–10% of flu cases in this group (Centers for Disease Control & Prevention, 2020a).

For COVID-19, the World Health Organization communicated both risks clearly, stating on its website in April 2020: “Evidence to date suggests that children and young adults are less likely to get severe disease” and “children and adolescents are just as likely to become infected as any other age group and can spread the disease” (World Health Organization, 2020c). However, as shown in Table 1, other messages are less clear about *what* is riskier for groups identified as “at increased risk.” It is notably unclear that this “risk” does not refer to the chance that one may become infected. When one reads that people over 70 years old are “at higher risk from coronavirus,” they may consider (as the message intends) that the elderly are more likely to be severely affected by COVID-19. They may also consider that the elderly are more likely to contract the disease (and become contagious)—which is not what the message intends. These risk messages could therefore be misunderstood as meaning that vulnerable people are more likely to be infected by the virus, in addition to, or instead of, being more likely to suffer severe symptoms because of it. We could therefore expect that the “at increased risk” messages create a difference in the perceived infection probabilities for vulnerable and nonvulnerable individuals. This could be because people simply raise their perceptions of the probability that a vulnerable individual would be infected. However, an alternative, nonexclusive, possibility is that people might lower their perceived probability that *nonvulnerable* individuals would be infected.

## Inferences of *Lower-Than-Usual* Risks to Nonvulnerable Groups

When risk communicators state that certain groups are “at increased risk,” the message is intended to mean that the disease is more dangerous for some individuals (because they are more likely to suffer severe symptoms from it). Semantically, the statement says nothing about the change of risk levels to nonvulnerable groups, and is presumably not intended to be interpreted as such. However, people often infer, pragmatically, meanings that go beyond the semantics of what communicators have explicitly said (Horn, 2006). Inferences drawn from speakers’ choices of words regularly shape the interpretation of language in general (e.g., Hilton, 2008; Ingram et al., 2014; Keren, 2007; Sher & McKenzie, 2006) and risk quantifiers in particular (Juanchich et al., 2020; Sirota & Juanchich, 2012).

Evidence suggests that when a particular risk is emphasized, people can infer that other, independent, risks are less likely to occur (Park et al., 2021; Windschitl et al., 2017). Therefore, when health authorities repeatedly communicate regarding the risks for vulnerable groups, this message could be taken as implicitly meaning: “the risk for other (nonvulnerable) people is lower than what one might expect,” rather than that risk simply being lower compared to the vulnerable group. At the start of the pandemic, when data was limited, any individual might have assumed that everyone faced equal risks from the virus. However, when faced with national health messages highlighting that “people who are 70 or older are at increased risk from coronavirus,” instead of simply adjusting upward the perceived risks to the vulnerable group described in the messages, people may also have inferred that younger adults were at less risk than initially perceived.

Because people regularly make pragmatic inferences, we hypothesized that exposure to risk messages that highlight higher risk to vulnerable groups would cause people to lower their perception of the risks to *nonvulnerable* individuals (instead of simply heightening their perceptions for vulnerable individuals). Lower perception of risks to nonvulnerable individuals could come in the form of lower perceived probability of severe symptoms or lower perceived probability of infection, depending on how the term “risk” is interpreted. We now know that the probability of severe symptoms for “nonvulnerable” individuals (the majority of people) is indeed lower than first expected (Verity et al., 2020), so this is not an inaccurate perception. However, believing that nonvulnerable individuals are less likely to *contract* COVID-19—whether compared to vulnerable individuals or compared to prior beliefs —is problematic because there is no consistent evidence that this is the case (e.g., Bi et al., 2020; Jing et al., 2020; Li et al., 2020). Earlier in the pandemic, some evidence suggested that COVID-19 case rates were lower in children (Stokes et al., 2020; Williams et al., 2021), which might suggest this was a nonvulnerable group for infection. However, evidence later emerged that children can, and are just as likely as adults, to be infected, even if they are less likely to develop symptoms (Zimmermann & Curtis, 2020). If frequent exposure to ambiguous “at risk” messages lowers perceived infection probability for nonvulnerable groups like children, this could lead to inaccurate risk perception because it would hinder people from adjusting their probability estimates upward to account for new knowledge. Further, the misinterpretation that one is less likely to be infected may reduce support for protective measures (Beale et al., 2021; Lewnard & Lo, 2020). The majority of the population can be classed as nonvulnerable, and it is important that they are able to accurately interpret risk messages and make appropriate inferences about their likelihood of contracting the virus so that they take appropriate protective measures to reduce transmission.

## Objectives of Research

We posited that the terminology “at increased risk from coronavirus” raises two concerns. First, people may be confused about whether the higher risk is referring to contracting the disease or to developing severe symptoms. Second, people could draw incorrect inferences about the relative magnitudes of each risk across groups compared to their prior expectation. We report five studies evaluating the ambiguity of risk messages that describe specific groups as being “at increased risk” from coronavirus and the inferences that people draw from these risk messages. We expected that some people would believe that being “at increased risk” meant being more likely to *contract* coronavirus and that this interpretation would affect their probability perceptions for vulnerable people to become infected by coronavirus. Furthermore, we expected that exposure to the risk message (vs. no exposure) would lead people to lower their estimated probabilities that nonvulnerable individuals would be infected. Finally, to provide an evidence-based solution to resolve the ambiguity of the term “risk,” we tested whether a clearer message that specifically mentioned exactly what risk is higher for vulnerable individuals would improve interpretations and probability perceptions (Experiments 4–5).

### Open Science Statement

The five studies were preregistered. The preregistrations, along with materials and data for all the studies, are shared on the Open Science Framework (https://osf.io/q78ax/). All studies received approval from the University of Essex’s research ethics committee prior to data collection.

## Study 1

Study 1 was conducted with U.K. residents recruited on Prolific on a single day (April 7, 2020). This was after the U.K. government had announced and enforced additional measures to control the COVID-19 pandemic (as of March 24, 2020): A stay-home order (including a ban on visiting other dwellings) with limited exemptions, closure of all except specified businesses and venues, and a ban on gatherings of more than two people in public spaces.

In Study 1, participants were first randomly allocated to see a risk message regarding vulnerable groups or not. They then provided probability estimates of infection and severe symptoms for both vulnerable and nonvulnerable individuals before providing their interpretation of the risk message.

We hypothesized that people would interpret that the term “risk” referred to the chance of developing severe symptoms (as intended by the message) but also believe that “risk” referred to the possibility of contracting the *infection* (which the message does not intend; H1.1). As a result, we expected that in addition to perceiving that vulnerable older adults had higher chances of hospitalization, participants would believe that they also had higher chances of infection compared to nonvulnerable others, and that this would be especially the case for people who believed that the term “risk” referred to probability of infection (H2.1). We also expected that exposure to the risk message (compared to no exposure) would increase the difference in estimated infection probability for vulnerable and nonvulnerable individuals because the message would lower the perceived probability that nonvulnerable individuals could become infected by the virus (H3.1). Finally, we expected that probability perceptions would be related to health recommendations participants would give to others (H4.1).

### Method

#### Participants

We recruited 396 participants (after excluding eight participants who failed an attention check, a preregistered exclusion criterium described below in the procedure). Participants were 56% female (43% male, 1% other or did not disclose), 79% White, and ages ranged from 18 to 79 years (*M* = 43.4, *SD* = 15.3 years). Further sociodemographic characteristics are reported in Table 2.[Table tbl2]

#### Design, Materials, and Procedure

We used a mixed design where we manipulated exposure to a risk message at the onset of the study between-subjects and vulnerability within-subjects. Participants were either exposed to a risk message or not (*n* = 198 each) before assessing the probability that someone would contract coronavirus and the probability that they would suffer severe symptoms from such an infection. In the vulnerability manipulation, the probability judgments focused on three individuals: An older adult over 70 (vulnerable), a healthy younger adult, and a healthy child (both nonvulnerable).

**Figure 1 fig1:**
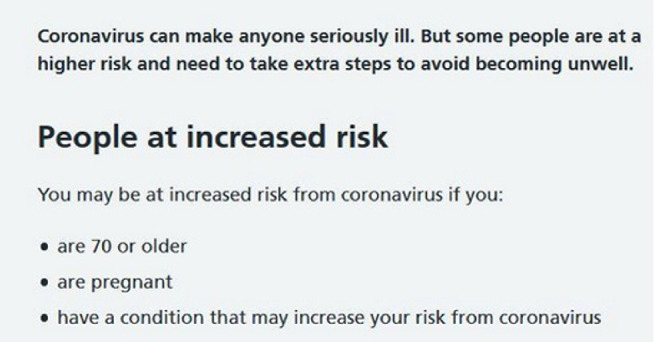
Risk Message Shown in the Experimental (Risk Message) Condition in Studies 1, 2, and 4 as Taken From the U.K. National Health Service (April 6, 2020) *Note*. See the online article for the color version of this figure.

The risk message was presented on a separate page from other questions as an image. The message was an image taken from the U.K. National Health Service (NHS) website and it identified the groups considered “at increased risk from COVID-19” (see Figure 1). In the experimental condition, participants read the message in Figure 1 and then proceeded to answer the questions about their probability perceptions, risk interpretation, and health recommendations. In the control condition, participants went straight to answering these questions.[Fig fig1]

#### Probability Perception Questions

On separate pages, participants evaluated the probability that three different individuals would be infected by the COVID-19 virus over the next 30 days and the probability that each of these individuals would require hospitalization if they were infected by the virus (see exact wording of the questions in Table 3). The three individuals were: A vulnerable individual who belonged to the group at increased risk (aged over 70 years) and two “nonvulnerable” individuals who did not (children, defined as younger than 18 years old, and adults aged between 18 and 50 years). Participants provided their estimates as a numerical probability (between 0 and 100%). Because the probability of hospitalization for coronavirus infection was conditional on already having coronavirus, the question about the chance of contracting coronavirus was always presented before the question about the chance of needing hospitalization after contracting coronavirus. Participants provided each estimate on a separate page, with order of presentation of the age groups randomized.^2^

#### Health Recommendations

After completing the probability perception questions, participants reported whether they would advise people from each of the three age groups to stay at home 24/7 over the next 14 days, using a 4-point scale (0: *not at all*, 4: *yes, completely*). This question was presented in a matrix table with all the age groups presented simultaneously.

#### Risk Message Interpretation

Participants then provided their interpretation of what the National Health Service meant when they advised that “some people are at increased risk of severe illness from coronavirus (COVID-19).” Participants could answer “Yes”, “No”, or “I do not know” for each of three interpretations (presented simultaneously in a matrix table in a set order, as in Table 4): Being in the higher risk group means having a greater chance of …

• carrying the virus.
• being infected by the virus.
• being hospitalized because of the infection.

[Table tbl4]

The first option was listed as a filler item so that participants would be less likely to perceive that the answers to the question were exclusive.^3^

Finally, participants provided sociodemographic information. Participants completed the online study at the end of a separate study asking about beliefs in conspiracy theories and health protective behaviors (Juanchich et al., 2021). The study included a preregistered attention check question (“Please select the option ‘definitely not true’ to show that you are reading the questions”) to detect poor response quality.

### Statistical Analyses

We tested preregistered hypotheses using planned analyses^4^ about interpretations and probability perceptions (H1.1–H1.3) using a multivariate analysis of variance (MANOVA) including vulnerability (vulnerable or nonvulnerable [children and younger adults together]), participants’ interpretation of the term “risk” (whether it meant chance of infection: “yes” vs. “no” and “do not know” combined), and message exposure condition (control or message) as fixed factors and probability perceptions for hospitalization and infection as dependent variables. The connection between probability perception and health recommendation (H4.1) was tested using correlational analyses. Statistical significance was determined at α = 0.05. For all effects involving variance analyses, we report partial η^2^ effect sizes.^5^ We report Cohen’s *d* effect sizes for pairwise group comparisons.

### Results and Discussion

#### Interpretation of the Term Risk and Associated Probability Perception

Most participants (95%) recognized that being at increased risk characterized the chance of being hospitalized (see Table 4). Correspondingly, the MANOVA found a main effect of vulnerability on probability perceptions: Participants perceived that older adults had higher chances of being hospitalized if they contracted coronavirus (*M* = 45.84%, *SD* = 30.82), compared to children and younger adults—the nonvulnerable individuals—on average (*M* = 16.58%, *SD* = 19.37), *F*(1, 392) = 601.88, *p* < .001, η_P_^2^ = 0.61. Interestingly, viewing the risk message did not raise the perceived probability that an older adult would be hospitalized in case of infection (compared to not seeing the message), *M*_message_ = 45.82%, *SD* = 30.97, *M*_control_ = 45.85%, *SD* = 30.75; *F*(1, 391) = 0.05, *p* = .832, η_P_^2^ < .001.

Supporting H1.1, over half our participants (56%) also interpreted “risk” to mean the chance of being *infected* with coronavirus (i.e., catching the disease), 95% CI [51%, 61%]. In line with this interpretation and supporting H2.1, participants perceived that older adults were more likely to become infected by the new coronavirus (*M* = 40.81, *SD* = 34.74) compared to younger adults and children taken together (*M* = 33.10, *SD* = 29.81), *F*(1, 392) = 85.03, *p* < .001, η_P_^2^ = 0.18. People who interpreted that “risk” meant the chance of infection were especially likely to believe that older adults had a higher chance of contracting the virus (*M* = 47.04, *SD* = 34.46) compared to people who did not interpret “risk” as chance of infection (*M* = 32.86, *SD* = 33.53), interaction effect: *F*(1, 392) = 35.19, *p* < .001, η_P_^2^ = 0.08. Participants also found it difficult to disentangle the probability of severe symptoms and the probability of infection, as indicated by the correlation between the two that we found for both children and younger adults together (nonvulnerable) and older adults (vulnerable), *r*_nonvulnerable_ = 0.50, *p* < .001; *r*_vulnerable_ = 0.55, *p* < .001.

#### Effect of Exposure to Risk Message on Inferences About Probability of Infection for Nonvulnerable Individuals

**Figure 2 fig2:**
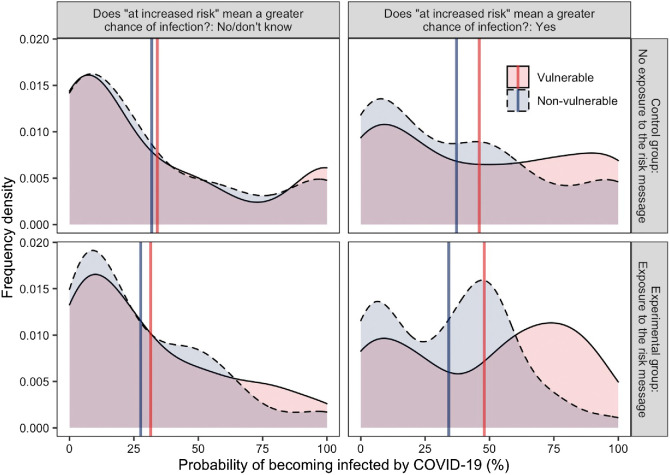
Participants’ Perceived COVID-19 Infection Probability for Vulnerable and Nonvulnerable Individuals in Study 1 *Note*. The figure shows perceived COVID-19 infection probabilities for vulnerable and nonvulnerable individuals as a function of participants’ interpretation that risk referred to the probability of infection (no/do not know, left panels, *n* = 174 vs. yes, right panels, *n* = 222) and as a function of the experimental message condition (control—no risk message, top panels, *n* = 198 vs. exposure to the experimental message, lower panels, *n* = 198). The shaded area shows the frequency density of responses. Solid vertical lines give the mean probability estimate in each condition. COVID-19 = coronavirus disease. See the online article for the color version of this figure.

As shown in Figure 2, the effect of exposure to the risk message on the perceived probability of infection varied as a function of the level of vulnerability of the individual for whom the judgment was made and as a function of participants’ interpretation of “risk” as chance of infection.[Fig fig2]

The MANOVA supported our hypothesis that exposure to the message would affect probability perceptions (H3.1), with an interaction effect between vulnerability and message exposure, *F*(1, 392) = 5.95, *p* = .015, η_P_^2^ = 0.02, and a significant three-way interaction between vulnerability, exposure to the risk message, and participants’ interpretation of “risk” as infection, *F*(1, 392) = 3.95, *p* = .047, η_P_^2^ = 0.01. People who did not endorse the “infection” interpretation (*n* = 174) had a similar probability perception for infection among both children and younger adults (nonvulnerable) and older adults (vulnerable) whether they saw the message or not (shown in the left panels of Figure 2, with the blue and red lines close and in a similar location for the top and bottom panels). However, participants who endorsed the “infection” interpretation (*n* = 222) believed that older individuals were more likely than the other individuals to be infected, and this tendency was stronger in the experimental condition: The estimated difference in the probability of infection between older adults and the other nonvulnerable individuals (as a group) was greater after exposure to the risk message, as indicated by the larger gap between the red and blue lines in the top right panel compared to the bottom right panel of Figure 2. Among participants who interpreted the “risk” as infection, a pairwise comparison showed that probability perceptions for nonvulnerable individuals (children and younger adults) was, as predicted, lower with exposure to the message than no exposure (*M*_message_ = 27.20, *SD* = 23.62; *M*_control_ = 34.64, *SD* = 33.59), but the effect was not statistically significant, *t*(188.81) = 1.90, *p* = .060, *d* = −0.26.

#### Probability Perceptions and Health Recommendations

Supporting H4.1, participants’ probability perceptions were significantly positively correlated with how much people advised younger individuals to stay home, for infection probability: *r* = 0.12, *p* = .012 (child), *r* = 0.12, *p* = .014 (younger adult); and for hospitalization probability: *r* = 0.43, *p* < .001 (child), *r* = 0.13, *p* = .010 (younger adult). Notably for children, the correlation was much larger for perceived hospitalization probability than perceived infection probability, showing that severity had more influence than likelihood on recommendations. However, probability perceptions for older adults were not correlated with advice to this group to stay home, infection probability: *r* = 0.09, *p* = .091; hospitalization probability: *r* = 0.01, *p* = .824. Here, there was possibly a ceiling effect for advice to stay home (*M* = 3.81, *SD* = 0.50 for a 4-point scale). Mean recommendations for children and younger adults were *M* = 3.07 (*SD* = 0.80) and *M* = 3.09 (*SD* = 0.71).

#### Interim Discussion

Participants understood that “being at increased risk” meant being more likely to be hospitalized, but half also believed that the risk referred to the possibility of *being infected* with the new coronavirus. Participants perceived that nonvulnerable individuals (e.g., younger adults and children) were less likely to be infected than vulnerable ones, and especially so when they misinterpreted “at increased risk from coronavirus” to refer to the chance of coronavirus infection (not just severe symptoms, as was intended). We also found that exposure to the risk message did not increase participants’ perception that vulnerable individuals could suffer severe symptoms from the illness. Instead, it affected participants’ perception of the probability of infection. For people who interpreted the “risk” as the chance of being infected, exposure to the risk message lowered their estimated probability of infection for nonvulnerable individuals and raised their estimated probability of infection for vulnerable ones (three-way interaction effect). This meant that participants who misinterpreted the “risk” as chance of infection had a larger gap in their probability perception of infection for vulnerable and nonvulnerable individuals.

## Study 2

In Study 2, we sought to replicate the findings of Study 1: The misinterpretation of the risk terminology and the effect of an ambiguous risk message on probability perceptions. We extended Study 1 by testing whether the effects would still hold when participants were assessing risks for only one group (vulnerable or nonvulnerable) instead of both as was the case in Study 1. Repeating Study 1 with a between-subjects design thus allowed us to rule out the possibility that participants estimated different probabilities for different age groups simply because they were asked to repeat the estimates (as evaluations can change depending on whether they are made jointly or separately; Hsee, 1996). Our hypotheses were the same as Study 1 (here numbered H1.2–4.2). The study was conducted with U.K. residents from Prolific on a single day (April 30, 2020). At this point, the U.K. still had in place the same measures to limit the spread of COVID-19 as in early April 2020 when Study 1 was conducted. By this time, the NHS had also updated its “at increased risk” message to highlight three vulnerable groups of people: 70 years or older, pregnant, or with a condition that might increase risk.

### Method

#### Participants

We recruited 399 participants (after excluding seven participants who failed an attention check). Participants were 62% female (37% male, 1% other or did not disclose), 88% White. Ages ranged from 18 to 71 years (*M* = 35.1, *SD* = 12.6 years). Further sociodemographic characteristics are reported in Table 2.

#### Design, Materials, and Procedure

The materials and procedure were identical to Study 1, except participants only provided one set of probability perception judgments and recommendations, either for children or for older adults. Participants were randomly assigned to one of four between-subjects conditions, which came from crossing the message manipulation from Study 1 (exposure to the risk message, *n* = 199, or not, *n* = 200) and the age of the individual for whom participants provided their probability perceptions and recommendations (children [nonvulnerable], *n* = 200, or older adults [vulnerable], *n* = 199). We focused on children for the nonvulnerable group because this was where the effect of the message was largest in Study 1, thus affording us more power to detect it while reducing the number of possible comparisons in the analysis.^6^ The risk message was identical to Study 1. Study 2 was also completed online, at the end of a separate study similar to that in Study 1.

### Statistical Analyses

We had the same analytical approach to test our hypotheses as in Study 1, except that vulnerability (vulnerable vs. nonvulnerable) was now entered as a between-subjects factor in the MANOVA.

### Results and Discussion

#### Interpretation of the Term Risk and Associated Probability Perception

Most participants (96%) recognized that being at increased risk characterized the chance of being hospitalized (see Table 4). Consistently, participants perceived that older adults had a higher probability of severe symptoms due to COVID-19 (*M* = 46.03%, *SD* = 28.44%) than children (*M* = 13.41%, *SD* = 20.23%), *F*(1, 391) = 170.71, *p* < .001, η_P_^2^ = 0.30. Exposure to the risk message did not affect participants’ perception of hospitalization probability or the difference in hospitalization probability perception across the two groups, *F*(1, 391) = 0.68, *p* = .411, η_P_^2^ < 0.01 (main effect); *F*(1, 391) = 0.18, *p* = .672, η_P_^2^ < .01 (interaction effect).

**Figure 3 fig3:**
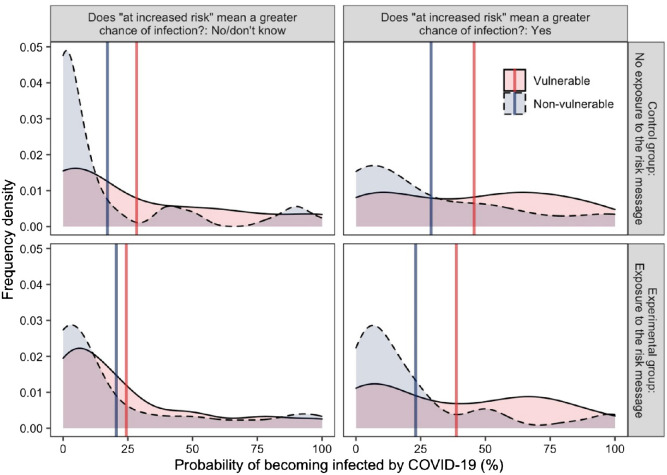
Participants’ Perceived Probability of Infection From COVID-19 in Study 2 *Note*. The figure shows perceived COVID-19 infection probabilities for vulnerable and nonvulnerable individuals as a function of participants’ interpretation that risk referred to the probability of infection (no/do not know, left panels, *n* = 179 vs. yes, right panels, *n* = 220) and as a function of the experimental message condition (control—no risk message, top panels, *n* = 200 vs. exposure to the experimental message, lower panels, *n* = 199). The shaded area shows the frequency density of responses. Solid vertical lines give the mean probability estimate in each condition. COVID-19 = coronavirus disease. See the online article for the color version of this figure.

Again, supporting H1.2, a majority of participants (55%) believed that the term “risk” referred to the chance of being infected with coronavirus, 95% CI [50%, 60%]. Overall, participants perceived different probabilities of infection for vulnerable and nonvulnerable individuals (as shown by the gap between the blue and red vertical lines in Figure 3). Supporting H2.2, participants judged that older adults were more likely to contract coronavirus than children, *F*(1, 391) = 13.99, *p* < .001, η_P_^2^ = 0.04. Probability perception was also shaped by participants’ risk interpretation (left vs. right panels of Figure 3): Participants who responded that “risk” referred to the chance of infection perceived a higher probability of infection for both groups (compared to participants who did not interpret risk that way), *F*(1, 391) = 13.20, *p* < .001, η_P_^2^ = 0.03. The difference in probability perception for the two groups (children [nonvulnerable] vs. older adults [vulnerable]) was slightly larger in people who believed risk referred to chance of infection (compared to people who did not), but this interaction term was not statistically significant, *F*(1, 391) = 1.84, *p* = .176, η_P_^2^ = 0.01.[Fig fig3]

#### Effect of Exposure to Risk Messaging on Inferences About Probability of Infection for Nonvulnerable Individuals

We expected to replicate Study 1, where exposure to the risk message increased the gap between the perceived probability of infection for children and older adults by increasing probability perception for older adults and decreasing it for children (H3.2). However, the trends shown in Figure 3 showed that participants exposed to the message (compared to those who were not) lowered their probability perceptions for both groups. The analyses also did not support our expectation, as we did not find that exposure to the risk message interacted significantly with the interpretation of the risk message or the vulnerability of the person to predict infection probability perception, respectively: *F*(1, 391) = 0.92, *p =* .338, *F*(1, 391) = 0.43, *p* = .512. Exposure to the risk message did not have a main effect on risk perception either, *F*(1, 391) = 1.09, *p* = .609.

For comparison purposes with Study 1, we conducted an independent samples *t*-test evaluating the effect of exposure to the risk message on how participants who believed that “risk” referred to the chance of infection judged children’s probability of infection. This tested our hypothesis about unintended inferences more directly and showed at the descriptive level that the average difference was similar to Study 1 in direction and magnitude: Participants who were exposed to the risk message felt that children were 7% *less likely* to be infected by the virus compared to participants in the control group. However, this difference was not statistically significant, *t*(102) = 0.99, *p* = .324, *d* = −0.20.

#### Probability Perceptions and Health Recommendations

Advice to stay home was still high on average, especially for older adults (*M*_older adults_ = 3.43, *SD* = 0.66, *M*_children_ = 2.85, *SD* = 0.90). Supporting H4.2, participants’ advice for children to stay home was positively correlated with probability perceptions of infection and hospitalization, with a larger correlation with hospitalization probability, *r* = 0.19, *p* = .006; *r* = 0.25, *p* < .001. There was also a positive correlation between advice for older individuals to stay at home and probability perception of infection and hospitalization—again larger, and only statistically significant, for hospitalization probability, *r* = 0.08, *p* = .248; *r* = 0.17, *p* = .017.

#### Interim Discussion

Overall, Study 2 showed that the majority of our sample misinterpreted the “increased risk from coronavirus” as referring to the chance of infection in addition to (rather than only) the chance of severe symptoms. This was similar to the finding in Study 1. As with Study 1, Study 2 (with vulnerability group manipulation conducted between-subjects) also found that participants estimated that older adults had higher chances of infection than children, which indicated that the difference in probability perception previously observed was not simply due to participants repeating estimates in the within-subjects design of Study 1. However, Study 2 did not replicate the interaction effect found in Study 1, where exposure to a risk message increased participants’ perceived probability of infection for vulnerable adults and, critically, reduced it for nonvulnerable individuals among participants who interpreted “risk” as chance of infection. The effect sizes for exposure to the message on these participants’ infection probabilities for children were both small (*d* = −0.27 in Study 1 and *d* = −0.20 in Study 2) and we had a lower chance of detecting the effect in Study 2, where the vulnerability manipulation was between-subjects, reducing statistical power.^7^ In Study 3, therefore, we aimed to replicate the test of this hypothesis while scaling up the statistical power by using a larger sample and the original within-subjects design for age groups.

## Study 3

In this study, we hypothesized that people would misinterpret what is at increased risk in an ambiguous version of the risk message (focusing on the statement “some people are at increased risk from “COVID-19”, H1.3). We also hypothesized that this misinterpretation would lead people to infer that nonvulnerable individuals were less likely than vulnerable ones to be infected (H2.3), and that seeing the message (compared to not seeing it) would lead people to believe nonvulnerable individuals were less likely to be infected (H3.3). In line with the previous studies, we expected that probability perceptions would be related to health recommendations (H4.3). We used a within-subject design to have a greater statistical power to detect the effect of the message found in Study 1.

Study 3 was conducted with U.K. residents from Prolific on a single day (July 28, 2020). At this point, the U.K. government had lifted the lockdown measures set in March 2020: Outdoor gatherings were allowed for up to six different households (from June 13, 2020) and indoor ones for six people from up to two different households (from July 4, 2020). The government had also announced that from August 1, 2020, it would no longer provide support (e.g., deliveries of essential supplies) for vulnerable individuals to self-isolate.

### Method

#### Participants

We powered our sample based on our smallest hypothesized effect: The effect of exposure to the message (vs. no message) on probability perceptions of infection for children. We recruited 432 participants, which gave 90% power to detect a small effect size between two independent groups (Cohen’s *d* = 0.28, α = .05). Participants were 67% female (32% male, 1% other or did not disclose), 84% White. Ages ranged from 18 to 71 years (*M* = 33.2, *SD* = 12.0 years). Further characteristics are reported in Table 2.

#### Design, Materials, and Procedure

Participants completed the study online. Participants provided the probability of infection from coronavirus for a child and for an adult over 70 years old (see exact question wording in Table 3). Participants evaluated these probabilities for the child and the older adult on separate pages, in a counterbalanced order for each participant. We manipulated whether participants saw a risk message before completing the probability perception questions (*n* = 216 each group). We simplified the message and presented it as text, shown on the same page as the questions:^8^

REMINDER: Some people are at increased risk from COVID-19

People who are over 70 years of age or people with a preexisting medical condition are at higher risk from COVID-19.

Participants also completed the same risk interpretation question from Study 1, but did so either before or after the probability perception questions (random allocation). This allowed us to check whether their risk interpretation might have been a function of having seen the risk message or not.^9^

Participants then completed a health recommendation task. They evaluated whether they would advise a healthy 15-year-old child and an older adult who was 75 years old (presented in random order on the same page) to take three protective measures: Self-isolate at home, social distance at all times, wear a face mask whenever on any outing. Participants gave their recommendations on a 5-point scale anchored at “not at all” and “yes completely.” This scale was expanded to five points to mitigate the ceiling effect observed in Studies 1 and 2, where participants very largely agreed that older adults should “stay at home.” It included two additional protective measures not included in Studies 1 and 2, to reflect changes in the U.K. government’s guidance at the time of Study 3: “social distance at all times” and “wear a face mask whenever on any outing.” This accounted for the fact that by the time of Study 3, the stay at home order had been lifted and replaced by this advice. The scale had satisfactory reliability for both individuals (.61 and .72). Finally, participants provided sociodemographic information.

### Statistical Analyses

We analyzed the proportion of participants believing that the increased risk referred to the chance of infection (H1.3). To replicate the analyses from Studies 1–2, we ran analyses of variance (ANOVAs) on participants’ probability estimates, including as fixed factors vulnerability (vulnerable vs. nonvulnerable), exposure to the risk message (vs. no message), and risk interpretation (means infection vs. does not, and their interactions). We also ran preregistered group comparisons to specifically test H2.3 (the effect of risk interpretation on probability estimates for older adults) and H3.3 (the effect of the risk message on probability estimates for children). We tested H2.3 with an independent samples *t*-test comparing participants who interpreted “risk” as referring to the probability of infection by the virus versus those who did not. We tested H3.3 with an independent samples *t*-test comparing the perceived probability of children being infected for participants exposed to the risk message (compared to those who were not). Finally, we tested the link between probability perception and health recommendations (H4.3) with a correlational analysis as in the previous studies.

### Results and Discussion

#### Interpretation of the Term Risk and Associated Probability Perception

**Figure 4 fig4:**
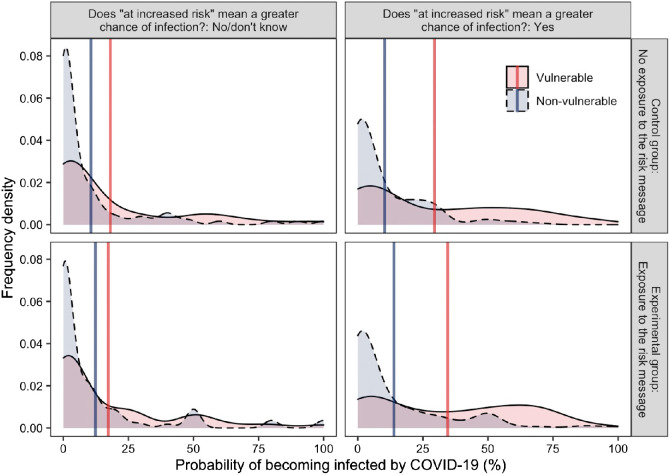
Participants’ Perceived Probability of Infection From COVID-19 in Study 3 *Note*. The figure shows perceived COVID-19 infection probabilities for vulnerable and nonvulnerable individuals as a function of participants’ interpretation that risk referred to the probability of infection (no/do not know, left panels, *n* = 169 vs. yes, right panels, *n* = 260) and as a function of the experimental message condition (control—no risk message, top panels, *n* = 216 vs. experimental exposure to the message, lower panels, *n* = 216). The shaded area shows the frequency density of responses. Solid vertical lines give the mean probability estimate in each condition. COVID-19 = coronavirus disease. See the online article for the color version of this figure.

As expected in H1.3, around 95% of participants interpreted that risk referred to the chance of severe symptoms requiring hospitalization, (95% CI [93%, 97%]; see breakdown in Table 4). More than half the sample believed that being “at increased risk” meant having an increased chance of being infected with coronavirus, 60%, 95% CI [56%, 65%]. The ANOVA found that overall, participants perceived older adults were more likely to be infected than children (as shown by the gap between the blue and red vertical lines in Figure 4), *F*(1, 425) = 152.99, *p* < .001, η_P_^2^ = 0.27. Risk interpretation also affected infection probability perception, with participants who thought there was an increased risk of infection (vs. those who did not) estimated higher probabilities of infection overall, *F*(1, 425) = 12.82, *p* < .001, η_P_^2^ = 0.03. The difference in infection probability perception for children versus for older adults was larger in people who believed risk referred to probability of infection (vs. people who did not), with a significant interaction between these variables, *F*(1, 425) = 41. 73, *p* < .001, η_P_^2^ = 0.09.[Fig fig4]

The independent samples *t*-test found that as hypothesized (H2.3), participants who interpreted “risk” as referring to the chance of infection (compared to those who did not) perceived a greater likelihood that older adults would be infected (*M*_risk is infection probability_ = 32.07, *SD* = 28.72; *M*_risk is not infection probability_ = 17.70, *SD* = 25.41), *t*(388.92) = 5.43, *p* < .001, *d* = 0.52.

#### Effect of Exposure to Risk Message on Inferences About Probability of Infection for Nonvulnerable Individuals

We expected that participants who saw the risk message would perceive that children were less likely to be infected compared to a no-message control condition, especially when they interpreted that “risk” referred to the chance of infection. However, as shown in Figure 4, this was not the case. The ANOVA did not find that exposure to the risk message had a significant main effect, nor any significant interactions with vulnerability nor a three-way interaction with risk interpretation and vulnerability, *F*(1, 425) = 1.32, *p* = .252, η_P_^2^ < 0.01; *F*(1, 425) = 0.05, *p* = .831, η_P_^2^ < 0.01; *F*(1, 425) = 0.93, *p* = .336, η_P_^2^ < 0.01, respectively.

The preregistered *t*-test of the effect of message on probability estimates for children was also not statistically significant, (*M*_message_ = 13.24, *SD* = 21.08, *M*_control_ = 10.49, *SD* = 16.89), *t*(410.44) = −1.50, *p* = .135, *d* = 0.14.

#### Probability Perceptions and Health Recommendations

Participants’ probability perceptions for infection were significantly positively correlated to protective health recommendations for children and older adults, *r* = .23, *p* < .001 and *r* = .22, *p* < .001.

#### Interim Discussion

Overall, Study 3 showed that, consistent with findings from Studies 1 and 2, more than half of the people surveyed misinterpreted the “increased risk from coronavirus” as the chance of infection rather than just the chance of severe symptoms. Across three studies, this interpretation was connected with a probability perception gap: The perception that nonvulnerable individuals (e.g., children) were *less* likely to contract COVID-19 than vulnerable individuals (e.g., adults over 70 years old). Study 4 proposed solutions to reduce this gap. However, in Study 3, we did not find evidence that participants who were exposed to the risk message (vs. those who were not) inferred that nonvulnerable individuals were less likely to become infected by the virus. This was in contrast with Study 1, where the message widened the gap in probability perception for the different groups, but consistent with Study 2. We suspected this might be related to high exposure to the same message outside of our studies and addressed this issue in Study 5.

## Study 4

Studies 1–3 pointed out the pitfalls of current communication strategies. In Study 4, we sought to provide a solution that addressed these pitfalls. We crafted an improved message that specifically mentioned what the increased risk referred to (i.e., “at increased risk of developing severe symptoms”). We expected that this would improve clarity as previous work indicated that risk messages were better understood when they were specific about risk events (Gigerenzer & Edwards, 2003; Gigerenzer et al., 2005; Gigerenzer & Galesic, 2012). We adapted the original risk message from Studies 1 and 2 to indicate the risk was for developing severe symptoms. We compared the new message to the original to test the hypothesis that the new message would counteract the misinterpretation that the risk was the probability of infection (H1.4). We also hypothesized that the new message (compared to the original) would reduce the discrepancy in probability perceptions that a vulnerable and nonvulnerable individual would become infected (H2.4). Study 4 was conducted with U.K. participants using Prolific on a single day (February 22, 2021). At this time, the U.K. had entered its third period of lockdown (since January 6, 2021), with all people to stay home except for limited reasons.

### Method

#### Participants

We recruited 474 participants, which gave 90% power to detect a small-to-medium effect size between two independent groups (Cohen’s *d* = 0.27, α = .05). Participants were 69% female (29% male, 1% other or did not disclose), 84% White. Ages ranged from 18 to 74 years (*M* = 34.51, *SD* = 11.86 years). Further sociodemographic characteristics are reported in Table 2.

#### Materials, Procedure, and Design

**Figure 5 fig5:**
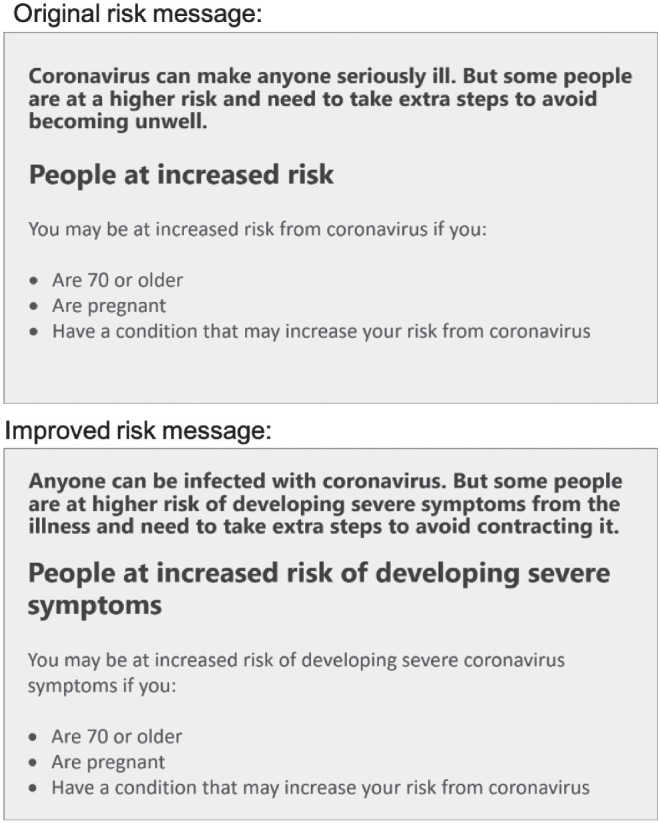
Original and Improved Risk Message With the Risk Event Clarified, Used in Study 4

Participants completed the study online. They were randomly allocated to view the original risk message from Studies 1 and 2 (*n* = 238) or an improved message that clarified the risk event (*n* = 236; see Figure 5). Participants read the text in the message on a separate page and then proceeded to the probability perception task, where the message always remained at the top of the page above two questions about the probability of infection and probability of hospitalization. We included both probabilities to check that the improved new message did not affect the probability perception for hospitalization. Participants estimated the probability of infection and hospitalization always presented in this order on the same page. They did these estimations for a child and for an adult over 70 on separate pages, with the order of presentation counterbalanced for each participant. Participants then proceeded to the risk interpretation question, in which they saw the risk message corresponding to their experimental condition and indicated their interpretation of the risk in the same way as in Studies 1–3. Finally, participants provided sociodemographic information.[Fig fig5]

### Statistical Analyses

We ran three confirmatory analyses to test our hypotheses about the effect of the improved risk message compared to the original message. First, to test H1.4, we used a χ^2^ test. Second, to test H2.4, we used an independent-samples *t*-test on the difference in probability perception between vulnerable and nonvulnerable individuals between message conditions. We also directly assessed the effect of the message on probability estimates using a mixed ANOVA on infection probability perception with vulnerability (within-subject), message (between-subjects), and their interaction as fixed factors.

### Results and Discussion

#### Does the Improved Message Reduce the Ambiguity?

As shown in Table 5, fewer participants interpreted the “risk” as the being about the chance of infection based on the improved message compared to the original message.^10^ However, H1.4 was not supported as this reduction was not significant, χ^2^(2, *N* = 474) = 4.94, *p* = .085.[Table tbl5]

#### Probability Perception of Contracting the COVID-19 Infection for Vulnerable and Nonvulnerable Individuals as a Function of Message Condition

**Figure 6 fig6:**
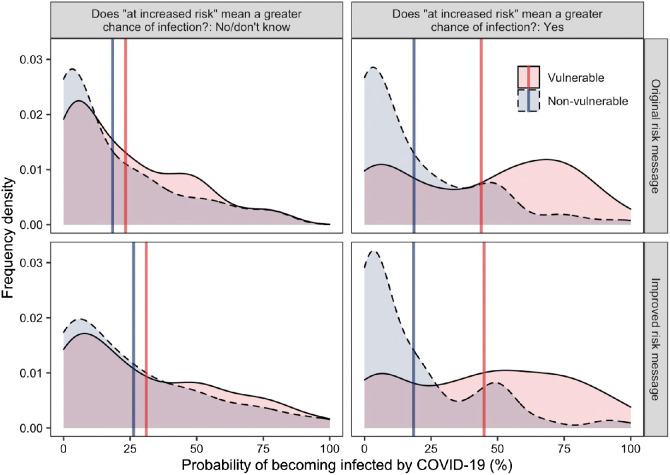
Participants’ Perceived Probability of Infection From COVID-19 in Study 4 *Note*. The figure shows perceived COVID-19 infection probabilities for vulnerable and nonvulnerable individuals as a function of participants’ interpretation that risk referred to the probability of infection (no/do not know, left panels, *n* = 240 vs. yes, right panels, *n* = 234) and as a function of the experimental message condition (original risk message, top panels, *n* = 238 vs. improved risk message, lower panels, *n* = 236). The shaded area shows the frequency density of responses. Solid vertical lines give the mean probability estimate in each condition. COVID-19 = coronavirus disease. See the online article for the color version of this figure.

As shown in Figure 6, on average, participants judged adults over 70 to be more likely to contract the virus than children. The pattern was similar in the original risk message as well as in the improved risk message condition. Older adults were on average perceived as 15% more likely to be infected than children based on both the original and the improved message, *M*_original message_ = 15.60%, *SD* = 26.79%; *M*_improved message_ = 14.79%, *SD* = 25.90%), *t*(472) = 0.33, *p* = .739, *d* = −0.03.[Fig fig6]

We expected that the improved message compared to the original would increase the perceived probability of infection of children and would reduce that of older adults. This was the case for children but contrary to H2.4, the same was also true for older adults, but in the ANOVA, neither the main effect of the message nor its interaction with vulnerability were statistically significant, *F*(1, 472) = 3.04, *p* = .082, η_P_^2^ = 0.01, *F*(1, 472) = 0.11, *p* = .739, η_P_^2^ < .001. The new message also had no detrimental effect on the perceived probability of hospitalization compared to the original, as older adults were overall still perceived to have a higher chance than children to be hospitalized, *F*(1, 472) = 989.97, *p* < .001, η_P_^2^ = 0.68, with the message having no effect on this perception difference, *F*(1, 472) = 1.89, *p* = .169, η_P_^2^ = 0.004, nor a main effect on hospitalization probability perception, *F*(1, 472) = 3.19, *p* = .075, η_P_^2^ = 0.01.

#### Infection Probability Perception as a Function of Risk Interpretation

As shown in Figure 6, we found that people who interpreted “risk” as chance of infection (compared to those who did not) showed a much wider gap in probability perception between children and older adults, which was supported by a significant two-way interaction between risk interpretation and vulnerability, *F*(1, 470) = 88.75, *p* < .001, η_P_^2^ = 0.16. Compared to people who did not endorse the infection interpretation, those who endorsed it believed that older adults were significantly more likely to be infected, but there was only a nonsignificant numerical difference in the belief that children were less likely to be infected, *t*(453.29) = 6.48, *p* < .001, *d* = 0.60 and *t*(472) = 1.84, *p* = .067, *d* = −0.17. The improved message did not have a significant effect on changing the gap in probability perceptions between older adults and children, *F*(1, 470) = 3.76, *p* = .053, η_P_^2^ = 0.01. The interaction effect of message and risk interpretation was also not significant, *F*(1, 470) = 2.99, *p* = .085, η_P_^2^ = 0.01.

#### Interim Discussion

Study 4’s results replicated that people who misinterpreted “risk” as chances of infection exhibited a wider gap in infection probability perception for children and older adults. The improved message that explicitly stated the “risk” was of severe symptoms showed, descriptively, more intended interpretations that this risk referred to the probability of severe illness and not the probability of infection, but this improvement was not statistically significant. The new message also did not significantly affect probability perceptions. These results may indicate that the message was still not sufficiently improved, or that even when a message explicitly explained what is at risk, the term “risk” remained ambiguous, or that people had internalized the unintended interpretation that “risk” in the context of COVID-19 could refer to infection due to the repeated use of this ambiguous risk message over the past year. This possibility was tested in Study 5 by focusing on a new hypothetical context.

## Study 5

In the four studies reported above, we showed that U.K. residents misunderstood the health authorities’ “at increased risk” message as meaning a higher chance of being infected, not just of developing severe symptoms. However, these studies brought mixed evidence about whether messages focusing on the risk to vulnerable individuals could decrease the perceived risk to others—the nonvulnerable majority. While participants perceived nonvulnerable individuals were less likely than vulnerable individuals to be infected, we found no causal evidence that this was because of the ambiguity of the risk message. Being exposed to the risk message did not significantly reduce people’s perception of how likely children would be infected compared to a no-message (control) condition in Studies 2 and 3, although it did in Study 1. This inconsistency possibly occurred because of repeated exposure to this risk message throughout the pandemic. Aligned with this interpretation, we noted that the average perceived probability that children would be infected in the control conditions decreased over time from 29% in Study 1 (7 April), 23% in Study 2 (30 April), to 10% in Study 3 (28 July). In Study 5, therefore, we introduced a new hypothetical epidemic context. We hypothesized that in this novel context, an “increased risk” message would lead participants to misinterpret the risk, but improving the risk message could decrease the ambiguity in interpretation (H1.5). We also hypothesized that an “at increased risk” message compared to no message would lead participants to perceive a higher probability of infection for vulnerable individuals and a lower probability for nonvulnerable individuals, thereby causing a probability perception gap about the risk of infection (H2.5). However, we hypothesized that an improved risk message compared to an ambiguous message could reduce this gap (H3.5).

### Method

#### Participants

We recruited 454 participants; the sample size determined by a priori power analysis needed to detect a medium effect size of *d* = 0.34 (and assuming α = .05, 1−β = 0.90) in a two-group comparison between a control and an experimental condition (approximately *n* = 151 per group). Participants’ ages ranged from 17 to 71 years (*M* = 34.39, *SD* = 12.35 years). Participants were 69% female (30% male, 1% other), 82% White, and 54% had a university degree.

#### Design, Materials, and Procedure

**Figure 7 fig7:**
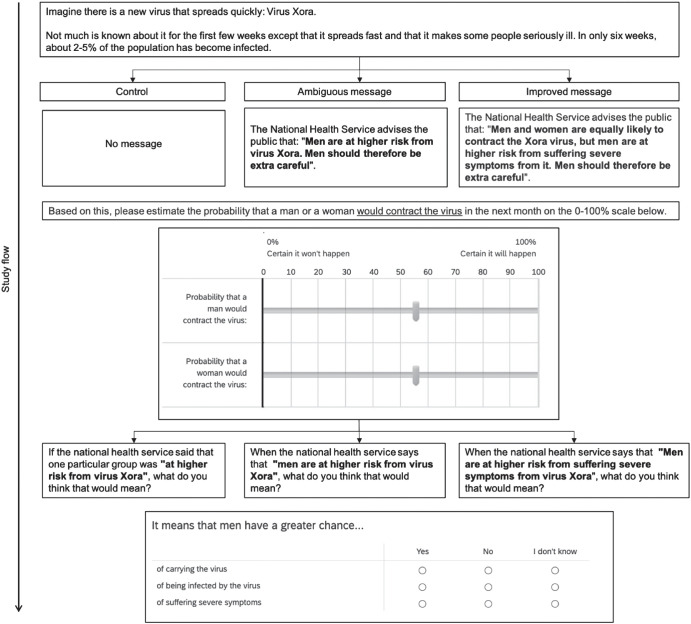
The Hypothetical Scenario, Control and Experimental Conditions, and Exact Questions Used in Study 5

Participants were randomly allocated to one of three conditions: A control condition or two experimental conditions, depicted in Figure 7. Participants read a basic scenario about a hypothetical new “virus Xora.” In the control condition (*n* = 152), participants were not exposed to any risk communication message with the scenario. In the two experimental conditions, the basic scenario was accompanied by a risk communication message that described men as being “at increased risk” from this new virus, which was either an ambiguous message (*n* = 151) or an improved message that specified the risk of severe symptoms (*n* = 151). These messages are shown in Figure 7. After reading this information, participants assessed the probability that a man or a woman would contract the hypothetical new “Virus Xora” on the same page as the scenario (and risk message in the experimental conditions) using the response scales shown in Figure 7. After reading this information, participants assessed the probability that a man or a woman would contract the hypothetical new “Virus Xora” on the same page as the scenario (and risk message in the experimental conditions) using the response scales shown in Figure 7.[Fig fig7]

Participants in all conditions subsequently provided their interpretation of what was more likely to happen when people were “at increased risk,” as described in the risk message (shown in Figure 7). This risk interpretation measure was the same as Studies 1–4. Finally, participants completed sociodemographic information. Participants completed the online study at the end of a separate study with other scenarios (e.g., estimating the likely costs of a road project, judging a hypothetical GP visit).

### Statistical Analyses

We used a χ^2^ test for H1.5, that fewer people would misinterpret “risk” as chance of infection when exposed to the improved message compared to the ambiguous message and control conditions. To test the role of the risk messages on participants’ infection probability estimates (H2.5–3.5), we first tested in the control group whether participants perceived men and women as equally likely to contract the new virus using a paired-samples *t*-test. To test our hypotheses that the risk message would impact probability perception, we first conducted an ANOVA using message condition (between-subjects), vulnerability (within-subjects), and their interaction as fixed factors. Then, to more specifically compare the different vulnerable groups, we conducted independent samples *t*-tests comparing participants’ infection probability estimates for men and women between the three message conditions.

### Results and Discussion

#### Effects of the Risk Messages on Risk Interpretation

In the control (no message) and the ambiguous risk message condition, 66%–74% of participants believed the risk referred to the probability of infection (see Table 6). In contrast, based on the improved risk message, participants’ interpretations were more consistent and only a minority (19%) endorsed the interpretation that being at “increased risk” meant having a greater probability of being infected by the virus (see Table 6). Indeed, supporting H1.5, the participants exposed to the improved message endorsed the “infection” interpretation significantly less often than those who saw no message or an ambiguous message, χ^2^(*N* = 303, d*f* = 2) = 78.71, *p* < .001 and χ^2^(*N* = 302, d*f* = 2) = 99.61, *p* < .001, respectively.[Table tbl6]

#### Infection Probability Estimates as a Function of Risk Message and Gender

Table 7 summarizes the differences in participants’ perception of the probability that men (vulnerable) and women (nonvulnerable) would become infected, as a function of the risk message. Participants perceived men to be more likely than women to be infected by the virus across all conditions, but this was more pronounced in the two experimental conditions that described men as “more at risk.” Indeed, the ANOVA showed a significant main effect of gender and interaction effect between gender and message condition, *F*(1, 451) = 122.21, *p* < .001, η_P_^2^ = 0.21; *F*(2, 451) = 40.10, *p* < .001, η_P_^2^ = 0.15, respectively.[Table tbl7]

**Figure 8 fig8:**
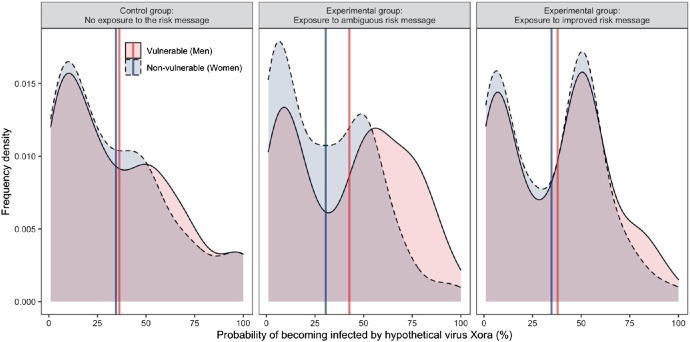
Participants’ Perceived Infection Probability From Hypothetical Virus Xora *Note*. The figure shows perceived infection probabilities for vulnerable (men) and nonvulnerable (women) individuals as a function of the experimental message condition (control—no risk message, left panel, *n* = 152 vs. ambiguous risk message, middle panel, *n* = 151 vs. improved risk message, right panel, *n* = 151). The shaded area shows the frequency density of responses. Solid vertical lines give the mean probability estimate in each condition. See the online article for the color version of this figure.

Our *t*-tests of the key comparisons showed that based on the ambiguous message (middle panel of Figure 8), participants perceived men were more likely to be infected than women (+16%), *t*(150) = 10.02, *p* < .001, *d* = 0.45. Based on the improved message (rightmost panel of Figure 8), participants still believed that men were more likely to be infected, but the difference was smaller and similar to that in the control condition (leftmost panel of Figure 8, +3%), *t*(150) = 4.43, *p* < .001, *d* = 0.12.[Fig fig8]

As expected in H2.5, compared to the control condition, the ambiguous message showed, descriptively, that participants believed that men were more likely to be infected by the virus (+6%) and that women were less likely to be infected (−4%), but these differences were not statistically significant, *t*(301) = 1.91, *p* = .057, *d* = 0.22 and *t*(294) = −1.31, *p* = .191, *d* = −0.15. Finally, as expected in H3.5, the improved (compared to the ambiguous) message showed, descriptively, a decrease in the perception that men would be infected by the new virus (−5%) and increase in the perception that women would be infected (+4%), however, these differences were not statistically significant, *t*(300) = −1.48, *p* = .139, *d* = −0.17, and *t*(300) = 1.47, *p* = .142, *d* = 0.17.

#### Interim Discussion

In Study 5, we used a hypothetical new virus with arbitrarily assigned vulnerable groups to test whether at the start of a pandemic, ambiguous risk messages would affect people’s interpretations of what was at risk and their subsequent probability perceptions of infection. We found the expected effect on interpretations: 74% of participants who viewed the ambiguous message (similar to the ones for COVID-19) interpreted “risk” as the chance of infection, but this was reduced to 19% among those who saw an improved message that specified that the risk was about suffering severe symptoms. Compared to the control and improved message conditions, exposure to the ambiguous message (like those used by various authorities at the beginning of the COVID-19 pandemic) also led to a larger difference in perceived infection probability between a vulnerable and nonvulnerable individual: Participants believed nonvulnerable individuals had a higher chance of infection than nonvulnerable ones. However, the pairwise comparisons only found small and nonsignificant evidence that participants perceived a nonvulnerable individual’s infection probability to be lower after seeing an ambiguous message relative to the control (*d* = −0.15) and improved message (*d* = −0.17).

## General Discussion

In five studies, we investigated two issues concerning the terminology “at increased risk,” which is often used to describe epidemic risks, and tested how to remedy these issues. We tested if people were confused about whether the risk referred to becoming infected or developing severe symptoms from an infection, and if this confusion led to the perception that vulnerable groups were more likely to be infected. We also tested whether “at increased risk” messages unintentionally lowered (compared to one’s baseline perception) the perceived probability that individuals not classed as vulnerable would be infected.

### Confusion About What Risk Is “Increased”

Two probabilities are critical in responding to a pandemic: The probability of becoming infected (related to how easily the virus spreads) and the probability of suffering severe symptoms because of the infection (related to how consequential the virus is). With the coronavirus pandemic, some groups are more likely to suffer severe symptoms, but there are no intrinsic characteristics that predispose groups to contracting the infection, and therefore everyone needs to adopt appropriate behaviors to avoid contracting and spreading the infection (WHO, 2020b). Across the world, health organizations have aimed to protect the most vulnerable (e.g., older individuals or those with long-term medical conditions) by explaining that they are “at higher risk” from COVID-19—meaning that they are more likely to develop severe symptoms. In this work, we posited that the term “risk” is ambiguous in this context because it can be taken as referring to either the probability of severe symptoms or to the probability of infection. Our findings show that most U.K. residents recognized that the term risk referred to the probability of severe symptoms, but half of them also believed that it referred to the probability of infection. This inconsistent interpretation of the higher COVID-19 risk highlights the importance of clearly identifying what a risk refers to and is in line with prior research on risks related to other medical conditions or even more ubiquitous events such as weather forecasts (Fischhoff et al., 2009; Gigerenzer & Galesic, 2012). In Study 4, we tried to improve the risk message to reduce the misinterpretation of “risk” as the probability of infection. Although fewer individuals who saw this message believed the risk was of infection (46%), this was not significantly less than participants who saw the original message (53%). However, in the context of a new hypothetical illness (Study 5), an improved message did significantly reduce the misinterpretation that “higher risk” means a greater chance of being infected. The difficulty of correcting misunderstandings of risk in Study 4 could thus have stemmed from participants having already been frequently exposed to ambiguous communication about COVID-19 by that point.

Misinterpretations of what is at “risk” are consequential for probability perceptions. While overall, participants tended to believe that vulnerable individuals, such as older adults, had a higher probability of coronavirus infection than nonvulnerable ones, this was especially the case for participants who misinterpreted “risk” as the chance of infection. At first glance, this pattern does not seem very problematic if it leads to more caution for vulnerable people. However, the flip side of this result is the perception that nonvulnerable individuals—meaning most of the population—have a lower chance of coronavirus infection. With this perception, the nonvulnerable majority may be more reluctant to follow health protection guidance (Bruine de Bruin & Bennett, 2020). Our data also showed an overlap between interpreting the risk as the chance of infection and the chance of carrying the virus—thereby infecting others (i.e., contagion), hinting that nonvulnerable individuals could also be perceived as less likely to spread the virus than vulnerable individuals.

### Does Focusing on Vulnerable Groups Lure Nonvulnerable Individuals Into a False Sense of Safety?

In communicating risk, it is rarely the case that speakers intend for recipients to interpret information as no more and no less than what is communicated, because language is used pragmatically (Horn, 2006). Listeners interpret the meaning of words with reference to context (e.g., Moxey & Filik, 2010; Moxey & Sanford, 1993a) and expectations (e.g., Moxey et al., 2009; Moxey & Sanford, 1993b). As a result, speakers’ choices of words are believed to convey implicit pieces of information (e.g., Hilton, 2008; Keren, 2007; Sher & McKenzie, 2006; Windschitl et al., 2017). Building on this pragmatic approach, we expected that emphasizing that some people are “at increased risk” could be taken to mean that others are less at risk than previously believed. We focused on the problematic possibility that people would perceive nonvulnerable individuals (i.e., most of the population) to have a lower than expected chance of contracting the new coronavirus. Importantly, in our work, we found across studies that children (who are considered nonvulnerable) were persistently perceived to have lower infection probability than older adults (who are considered vulnerable), and this gap in probability perception was quite wide. What caused the gap was, however, less clear. We found mixed evidence that directly exposing participants to an “at increased risk” message caused a lower perceived probability of infection for children. On average, participants believed children were less likely to be infected following exposure to the message in Studies 1 and 2, but these differences were small and not statistically significant (Study 1: *d* = −0.27, Study 2: *d* = −0.20), and the effect was not replicated in Study 3 (*d* = 0.14, opposite direction).

There are several reasons why we only observed small and inconsistent effects of exposure to the message. In our experiments, we focused on children as an exemplar of nonvulnerable individuals, since evidence was clear and remained consistent over time that children were less likely than adults, especially older adults, to suffer severe COVID-19 symptoms. However, the evidence on the likelihood of infection was more mixed. Participants’ perceived difference in infection probability for children and older adults in particular could be largely driven by earlier reports that there were lower COVID-19 incidence rates among children (Stokes et al., 2020; Williams et al., 2021). This does not fully explain why further exposure to a message would widen the difference in probability in Study 1, but it may be that people’s knowledge and experience of the disease shapes their interpretation of messages about it. This interpretation subsequently affects probability perception, as we found that participants who interpreted the message to mean a higher risk of infection (compared to those who did not) perceived a greater difference in the probabilities that a child or older adult would be infected. If interpretations of risk messages are in part shaped by knowledge of the disease, this could also explain why the interpretations proved difficult to correct in Study 4.

Another nonexclusive possibility for the lack of effect of experimental exposure to the message is that participants had frequent exposure to the message in the media, meaning that participants in the control group would also have seen it outside of the experiment. All our participants might therefore have already internalized the messages, thus limiting the ability of message exposure within our experiment to affect participants’ perceived probabilities. Indirectly supporting this hypothesis, we found that over time, participants gave decreasing estimates of the probability that a child would be infected—presumably due to repeated exposure to risk messages (29% in early April, 23% in late April, and 10% in late July). This trend of lower infection estimates is at odds with the emerging evidence over this period that children’s susceptibility to infection was greater than initially believed (Zimmermann & Curtis, 2020). However, when we tested in Study 5 whether people expected that not being vulnerable to a new illness meant being less likely to become infected, we still found a small effect, similar to Studies 1 and 2 (Cohen’s *d* = −0.20), which was again not statistically significant. These findings could mean that the risk message does not have the hypothesized undesired effect of lowering risk perceptions for nonvulnerable individuals compared to a baseline, or that the effect is small. Future research focusing on larger sample sizes would be more appropriate to identify or rule out such a small effect. Evaluating its practical significance could also be important since risk perception is so pervasive and important for decisions that small effects can be consequential.

### Practical Consequences of Risk Perception for Safeguarding Behaviors

An accurate perception of one’s likelihood of contracting a virus or suffering severe symptoms from it are important for making good quality health decisions. We found that lower risk perceptions led to weaker health recommendations, especially for younger age groups. Focusing on the risk of severe outcomes plays an important role in promoting health behaviors in general (Brewer et al., 2007; Sheeran et al., 2014), and in particular for COVID-19 (Bruine de Bruin & Bennett, 2020; De Neys et al., 2020). In line with this research, we found that the risk of severe symptoms seemed to weigh particularly on people’s recommendations for children, in particular, to self-isolate.

However, a key danger of COVID-19 is its infectiousness and the need for people to stop the spread of coronavirus by protecting *others* and not just themselves from the possibility of severe symptoms. Perceptions about one’s likelihood of contracting the disease are therefore highly important for dealing with the pandemic over the longer term. Conflating the likelihood of infection with that of severe symptoms is problematic because it means people are making important decisions based on information that may not be correct. For example, if people believe that younger individuals are inherently less likely to catch the virus than older ones, they may underestimate their own potential to infect others and spread the virus—including to more vulnerable individuals. Those in nonvulnerable groups could also believe it less necessary to adhere to onerous social distancing guidelines if they perceive themselves as less at risk of catching the virus. Further, emphasizing the communication to vulnerable groups places the onus of protective behaviors—and potentially the blame—on them rather than on the majority, who are actually the most likely to infect others because of their greater numbers (e.g., only 13% of the U.K. population are over 70 years of age; UK Office for National Statistics, 2020). The perceptions of risks to different groups (vulnerable and nonvulnerable) influences public support for actions such as “shielding” and reopening schools, and can therefore impact public health decisions.

Disambiguating the risk of severe symptoms and risk of infection is also important for health communication about vaccines. Determining the efficacy of a vaccine is complex and may involve its ability to protect against infection, severe symptoms, or both (Hodgson et al., 2021). Some vaccines could reduce risk of severe symptoms despite not reducing the risk of infection (e.g., in the case of some new coronavirus variants; Roberts, 2021), so a blanket belief that both risks are similarly reduced would be erroneous. Yet people do believe that they can mix freely with others after being vaccinated (Syal, 2021), highlighting the need to communicate more clearly whether the chances of infection are indeed reduced, along with the reduction in chances of severe symptoms.

### How Can the Risks of Infection and Severe Symptoms be Clarified?

Our results highlight that the term “risk” is ambiguous, so any health message about risk should fully explain *what* is at risk. In the context of an infectious disease such as COVID-19, this means communicating clearly and separately about the risk of infection and the risk of severe symptoms. It is important to be clear that some groups may be inherently at increased risk of severe symptoms whereas all groups have an inherently equal chance to contract the illness; but behavioral or situational factors can cause an increased risk of infection (e.g., the nature of people’s work and/or housing situation). Our results show that communicating clearly at the start of an epidemic (Study 5) could help people better identify the intended risk event in the message. However, ambiguous communication, especially over a longer period of time, may result in misinterpretations of the risk event, which are not easily corrected (Study 4). Going forward, clearer messaging about infection versus severe symptom probabilities could be applied to communication about new risks, for example, those posed by newly emerging virus variants, to ensure these are better understood.

### Limitations and Further Research

Our research provides evidence that can inform the effective communication of the various risks of COVID-19 to the public by making what is at risk explicit and by addressing all groups involved—especially those more likely to spread the virus. However, several limitations should be considered in further research. Our studies focused on risk perception for different age groups because these were distinct categories for which all health services had communicated risks for and advice to at the time of the studies (such as in Table 1). However, other factors increase risks of severe symptoms (e.g., having a respiratory health condition) that apply across age groups. We would expect similar results if we asked participants to estimate the risks to people with preexisting health conditions versus those without, but it would be good to extend this research to test this specifically.

We also acknowledge that this research took place over an evolving pandemic situation where very little was known with certainty about the virus. Most of our studies were also conducted while the government directive was for people to stay at home. People’s risk perceptions were likely shaped by changing information in the news, on public health websites, and indeed by nationwide restrictions on movements.

## Conclusion

Effective risk communication requires people receiving information to interpret the message accurately. In the case of COVID-19, people need to be aware of the chances of two different events: The chance of being infected and the chance of suffering severe symptoms from the illness. Our studies highlight the consequence of ambiguous risk communication, where people interpret a message that some people are “at increased risk from COVID-19” to mean these people have a higher chance of infection in addition to suffering severe symptoms. Problematically, people who harbored this interpretation that an increased risk refers to the chance of infection perceived that nonvulnerable individuals were less likely to become infected by the new coronavirus than vulnerable older adults—resulting in a larger perceived difference in the chance of infection between vulnerable and nonvulnerable individuals. Future research should establish whether this belief could lead nonvulnerable individuals to incorrectly assume that their own chances of becoming infected are lower, and the extent to which viewing risk messages could aggravate or correct this probability perception. Nonetheless, our results show that communicating risks clearly at the start of a disease outbreak could reduce the interpretational ambiguity of risk messages.

## Supplementary Material

10.1037/xap0000416.supp

## Figures and Tables

**Table 1 tbl1:** Examples of “At Increased Risk” Messages About Coronavirus From Three National Health Authorities

Health authority	Coronavirus/COVID-19 risk message
National Health Service (U.K.)	Coronavirus can make anyone seriously ill. But some people are at a higher risk and need to take extra steps to avoid becoming unwell.
	*People at increased risk*
	You may be at increased risk from coronavirus if you:
	• are 70 or older
	• are pregnant
	• have a condition that may increase your risk from coronavirus
Centers for Disease Control and Prevention (U.S.)	Older adults and people who have severe underlying medical conditions like heart or lung disease or diabetes seem to be at higher risk for developing serious complications from COVID-19 illness.
Australian Government Department of Health	Advice for people most at risk
	See more information and advice for people most at risk, including:
	• Aboriginal and Torres Strait Islander peoples and remote communities
	• older people
	• people in aged care facilities
	• people with chronic conditions
	• people with disability
	• travelers
*Note*. The U.S. Centers for Disease Control and Prevention message is clear on what is “at increased risk” whereas the Australian version is more ambiguous, with the U.K. NHS message being in between. Information gathered as of April 30, 2020 (Australian Government Department of Health, 2020; Centers for Disease Control & Prevention, 2020b; UK National Health Service, 2020). Public authorities may have changed the wording on their websites since. COVID-19 = coronavirus disease.	

**Table 2 tbl2:** Detailed Sociodemographic Characteristics of Participants in Studies 1–4

Characteristic	Study 1, April 7, 2020 (*n* = 396)	Study 2, April 30, 2020 (*n* = 399)	Study 3, July 28, 2020 (*n* = 432)	Study 4, Feb 22, 2021 (*n* = 474)
Highest level of education				
Less than high school	2%	1%	1%	0.2%
High school diploma	36%	33%	38%	32%
Bachelor’s degree	40%	44%	38%	43%
Master’s degree	16%	16%	15%	19%
Other	4%	7%	8%	6%
Personal income				
≤£10,000	23%	29%	29%	24%
£10,000–£20,000	21%	18%	19%	21%
£20,000–£30,000	20%	18%	19%	22%
£30,000–£40,000	12%	13%	9%	14%
£40,000–£60,000	10%	7%	8%	7%
>£60,000	3%	4%	4%	4%
Did not disclose	10%	11%	12%	9%
Political preferences				
Labor	35%	37%	33%	37%
Conservative	28%	20%	18%	15%
Liberal democrat	9%	11%	7%	8%
U.K. independence party	2%	1%	1%	0.4%
Green party	7%	11%	10%	9%
Other party	4%	4%	5%	4%
No preference	12%	11%	19%	18%
Did not disclose	5%	6%	7%	8%
Employment				
Unemployed	32%	32%	33%	26%
Working from home due to COVID-19	32%	28%	18%	30%
Usually working from home	12%	9%	5%	8%
Working as normal	10%	10%	35%	26%
Not able to work (other reasons)	15%	21%	9%	11%
Health symptoms				
Experiencing COVID-19-like symptoms^a^	3%	2.5%	—	—
No COVID-19 symptoms	96%	96.5%	—	—
Did not disclose	1%	1%	—	—
Living restrictions			—	—
Practicing social distancing	75%	75%	—	—
Under self-quarantine	21%	17%	—	—
No restrictions	4%	8%	—	—
Risk category				
At higher risk from COVID-19	—	—	12%	15%
Not at higher risk from COVID-19	—	—	78%	77%
Not sure	—	—	8%	8%
Did not disclose	—	—	2%	1%
*Note*. — = Were not measured in a particular study. Sociodemographic variables for Study 5 (*n* = 454) are reported in the text. COVID-19 = coronavirus disease.				
^a^ It is not possible to confirm whether someone has COVID-19 without a medical test; these tests are not widely offered in the U.K., so participants were only able to self-report symptoms.				

**Table 3:** Wording of Questions for Probability Perception Measure in the Different Conditions in Studies 1–4

Question about infection probability (all studies)	Question about hospitalization probability given infection (studies 1, 2, and 4)
Could you please evaluate the chances that [*a child/a healthy adult/an older adult*] aged [X *years*] contracts the new coronavirus over the next 30 days?	Assuming that [*a child/a healthy adult/an older adult*] aged [X *years*] has contracted a coronavirus infection, could you please evaluate the chances that this [*child/person*] will need to be hospitalized (e.g., because of developing pneumonia caused by the virus)?
*Note*. Participants were also given the following instruction for how to provide their answer: “Your answer can range from 0% to 100% and can include up to three decimal places (e.g., enter 0.01% for a chance of 1 in 10,000).” Individuals and ages in the square brackets were either a child < 18 years, a healthy adult aged 18–50 years, or an older adult aged >70 years, depending on the condition in the study. For Study 1, the two younger age groups were grouped and averaged to represent “nonvulnerable” individuals.	

**Table 4 tbl4:** Participants’ Agreement With Different Interpretations of What the Term “Risk” Refers to in “at Increased Risk From Coronavirus” in Studies 1–3

Interpretation of “increased risk”	Study 1 (*n* = 396)			Study 2 (*n* = 399)			Study 3 (*n* = 432)		
	Yes	No	Do not know	Yes	No	Do not know	Yes	No	Do not know
Increased risk means a greater chance of being infected	56%	39%	5%	55%	39%	6%	60%	36%	4%
Increased risk means a greater chance of being hospitalized^a^	95%	3%	4%	96%	2%	3%	95%	3%	2%
Increased risk means a greater chance of carrying the virus.	22%	68%	10%	21%	68%	11%	24%	69%	6%
Of participants who select the infection interpretation:									
% that also selected the hospitalization interpretations	95%			96%			95%		
*Note*. All figures rounded to the nearest whole number. Participants responded to each of the three interpretations presented in a matrix table similar to the structure shown.									
^a^ Intended interpretation.									

**Table 5 tbl5:** Percentage of Participants Answering “Yes,” “No,” and “I Do not Know” to Three Different Interpretations of What “Risk” Means in the Risk Messages in Study 4

Interpretation of “increased risk”	Original message			Improved message		
	Yes	No	Do not know	Yes	No	Do not know
Increased risk means greater chance of being infected	53%	44%	3%	46%	53%	1%
Increased risk means greater chance of being hospitalized^a^	92%	4%	4%	93%	5%	2%
Increased risk means greater chance of carrying the virus.	25%	65%	10%	18%	72%	9%
Of participants who selected the infection interpretation:						
% that also selected the hospitalization interpretation	90%			88%		
*Note*. Participants responded to each of the three interpretations presented in a matrix table similar to the structure shown.						
^a^ Intended interpretation.						

**Table 6 tbl6:** Percentage of Participants Answering “Yes”, “No”, and “I Do not Know” to Three Different Interpretations of What “Risk” Means in an “at Increased Risk” Messages in Study 5

Interpretation of “increased risk”	Control (no message)			Ambiguous message			Improved message		
	Yes	No	Do not know	Yes	No	Do not know	Yes	No	Do not know
Greater chance of being infected by the virus.	66%	25%	9%	74%	19%	7%	19%	75%	5%
Greater chance of suffering severe symptoms^a^	85%	4%	11%	73%	9%	19%	93%	2%	5%
Greater chance of carrying the virus.	40%	38%	22%	45%	34%	21%	15%	75%	11%
Of participants who selected the infection interpretation:									
% that also selected the hospitalization interpretation	86%			71%			79%		
*Note*. Participants responded to each of the three interpretations presented in a matrix table similar to the structure shown.									
^a^ Intended interpretation.									

**Table 7 tbl7:** Mean Perceived Chance That a Man and a Woman Would Be Infected From Hypothetical Virus Xora in the Three Conditions in Study 5

Experimental condition	Mean perceived chance of infection (*SD*)		% diff from control		
	Men	Women	% Diff M–W	Men	Women
Control: No risk message (*n* = 152)	36.24% (29.25)	34.49% (28.64)	+2%	—	—
Ambiguous message: Men are “at higher risk” (*n* = 151)	42.64% (29.11)	30.49% (24.32)	+12%	+6%	−4%
Improved message: Equally likely to be infected, but men at higher risk of severe symptoms (*n* = 151)	37.85% (26.95)	34.69% (25.27)	+3%	+2%	−0.2%

**Table A1 tbl8:** Beta Coefficients in the Mediation Analysis for Study 1

	Children			Adults aged 18–50			Older adults		
Effect	β	*b* [95% CI]	*p*	β	*b* [95% CI]	*p*	β	*b* [95% CI]	*p*
Effect of risk message on probability perceptions (*a*)									
Infection	−0.24	−7.29 [−13.36, −1.21]	.019	−0.13	−4.14 [−10.48, 2.20]	.200	0.02	0.54 [−6.33, 7.42]	.876
Severe symptoms	−0.01	−0.15 [−4.38, 4.08]	.944	0.01	0.27 [−4.00, 4.55]	.900	−0.001	−0.03 [−6.13, 6.07]	.992
Direct effect on recommendations to stay home of:									
Risk message (*c*’)	−0.14	−0.12 [−0.27, 0.04]	.149	−0.11	−0.07 [−0.21, 0.06]	.290	0.01	0.004 [−0.10, 0.10]	.935
Probability perception (*b*)									
Infection	0.06	0.002 [−0.001, 0.004]	.309	0.08	0.002 [−0.001, 0.004]	.183	0.11	0.002 [−0.0001, 0.003]	.059
Severe symptoms	0.14	0.01 [0.001, 0.01]	.014	0.09	0.003 [−0.001, 0.007]	.098	−0.05	−0.001 [−0.003, 0.001]	.381
Indirect (mediated) effect of risk message on advice to stay home (*ab*)	—	−0.01 [−0.06, 0.03]	—	—	−0.01 [−0.03, 0.02]	—	—	−0.0001 [−0.01, 0.02]	—
Mediation by infection probability perception	—	−0.01 [−0.05, 0.01]	—	—	−0.01 [−0.03, 0.01]	—	—	0.001 [−0.01, 0.02]	
Mediation by severe symptoms probability perception	—	−0.001 [−0.03, 0.03]	—	—	0.01 [−0.02, 0.02]	—	—	−0.0003 [−0.01, 0.01]	
*Note*. A negative coefficient reflects lower probability perceptions and less advice to stay home for the risk message compared to the control group where participants did not see the message. Letters reflect the pathways in the mediation model illustrated in Figure A1.									

**Table A2 tbl9:** Beta Coefficients in the Mediation Analysis in Study 2

	Child			Older adult		
Effect	β	*b* [95% CI]	*p*	β	*b* [95% CI]	*p*
Effect of risk message on probability perceptions (*a*)						
Infection	−0.08	−2.55 [−11.02, 5.93]	.554	−0.13	−4.20 [−13.40, 4.99]	.369
Severe symptoms	−0.05	−1.00 [−6.64, 4.64]	.727	−0.12	−3.49 [−11.43, 4.44]	.386
Direct effect on recommendations to stay home of:						
Risk message (*c*’)	0.25	0.22 [−0.02, 0.46]	.067	−0.10	−0.06 [−0.24, 0.12]	.526
Probability perception (*b*)						
Infection	0.14	0.004 [−0.0003, 0.01]	.067	−0.002	0.00001 [−0.003, 0.003]	.984
Severe symptoms	0.19	0.01 [0.002, 0.01]	.013	0.18	0.004 [0.0004, 0.008]	.032
Indirect (mediated) effect of risk message on advice to stay home (*ab*)	—	−0.02 [−0.09, 0.05]	—	—	−0.01 [−0.06, 0.02]	—
Mediation by infection probability perception	—	−0.01 [−0.06, 0.03]	—	—	0.0001 [−0.02, 0.03]	—
Mediation by severe symptoms probability perception	—	−0.01 [−0.07, 0.04]	—	—	−0.01 [−0.06, 0.02]	—
*Note*. A negative coefficient reflects lower probability perceptions and less advice to stay home for the risk message compared to the control group where participants did not see the message. Letters reflect the pathways in the mediation model illustrated in Figure A1. COVID-19 = coronavirus disease.						

**Figure A1 fig9:**
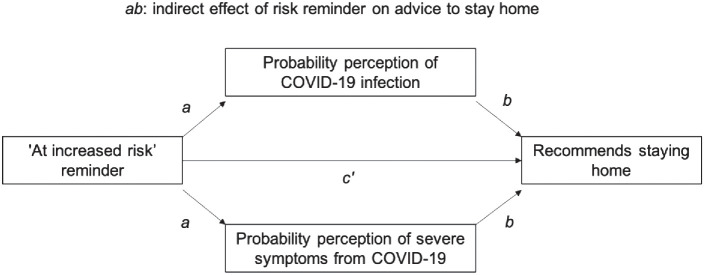
Mediation Model Testing the Effect of the “at Increased Risk” Message (vs. Control) *Note*.  The model tested the effect of the message on participants’ recommendations to others to stay home, as mediated by their probability perceptions for coronavirus infection and severe symptoms. The full set of coefficients for the pathways in the model are reported in Tables A1–A2.

## Footnotes

1 The World Health Organization defines “coronavirus” as the novel virus that causes the disease, which is known as “COVID-19” (World Health Organization, 2020a).

2 We also checked for two possible order effects. First, we tested whether presenting the infection probability question first or second (after the hospitalization question) had an effect on probability perceptions for each age group. There were no order effects on infection or hospitalization estimates for younger adults: *M*_infection_ = 33% versus 36%, *t*_infection_(394) = 0.96, *p* = .337; *M*_hospitalization_ = 16% versus 20%, *t*_hospitalization_(259.71) = 1.83, *p* = .068. Second, we tested the effect of presentation order of the two vulnerable versus nonvulnerable groups on probability perceptions. Participants who gave estimates for children first (compared to giving them later, after another age group) perceived higher infection and hospitalization estimates: For infection, *M* = 32% versus *M* = 22%, *t*(210.96) = −2.64, *p* = .009; for hospitalization, *M* = 17% versus 12%, *t*(216.19) = −2.12, *p* = .035. Participants who gave estimates for older adults first (compared to giving them later, after another age group) perceived lower infection estimates (*M* = 33% vs. *M* = 45%), *t*(394) = 3.49, *p* = .001, but no order effects were observed for hospitalization estimates, *M* = 44% versus 47%, *t*(394) = 0.87, *p* = .385. Note that these figures always reflect a higher probability estimate for older adults than younger adults and children, irrespective of which estimate was given first.

3 21% of participants considered the risk referred to carrying the virus, but all of them believed this was in addition to one of the other risk interpretations. We include a breakdown of participants’ who said yes to the “carrying the virus” option and an analysis of its effect on probability perception in Supplementary Material on the OSF (https://osf.io/q78ax/).

4 These hypotheses were preregistered, but numbered in a different way. The preregistered analyses also included mediation tests for the effect of the message on recommendations to stay home via probability perceptions using PROCESS in SPSS (Hayes, 2013). The mediation analysis is reported in full in the Appendix and showed that there were effects of the message condition on the perceived probability of infection to children and that these perceptions were positively related to health recommendations, but no mediation was found.

5 In an analysis of variance, partial eta-square (η_P_^2^) describes the proportion of variability associated with an effect after partialing out the variability associated with other effects in the analysis (Fritz et al., 2012).

6 For each of the two nonvulnerable age groups (separating children and young adult), exposure to the message lowered infection probability perception (compared to nonexposure), but that difference was only statistically significant for children, for children: *M*_*message*_ = 21.80, *SD* = 26.33, *M*_*control*_ = 28.97, *SD* = 34.47), *t*(369) = 2.32, *p* = .021, *d* = -0.23. and for young adult: *M*_*message*_ = 32.90, *SD* = 27.85, *M*_*control*_ = 37.04, *SD* = 35.85), *t*(371) = 1.28, *p* = .200, *d* = -0.13.

7 In Study 2, only 51 participants who viewed the message also believed the risk referred to the chance of infection, compared to 115 participants in Study 1.

8 We simplified the message and presented it on the same page as the questions to increase its salience while participants made their judgments.

9 The presentation order had no significant effect on the proportion of participants’ responses for each of the three interpretations of “at increased risk”: chance of infection, χ^2^(2) = 3.51, *p* = .173; chance of hospitalization, χ^2^(2) = 1.13, *p* = .570; chance of carrying the virus, χ^2^(2) = 2.59, *p* = .274. We also checked whether the presentation order affected probability perceptions; there was no effect on probability perceptions for either group, or the effects of the other independent variables, all *p*s > .10.

10 Only 0.6% of the sample indicated they did not know whether the risk was of being infected or being hospitalized; these participants were all in the original message condition.

## Results of Mediation Analyses in Studies 1 and 2

We report the results of two mediation analyses in Studies 1 and 2 that tested the hypothesis that differences in perceived risks due to viewing an “at increased risk” message would mediate recommendations to undertake protective health behaviors (e.g., self-isolating, social distancing). The model is depicted in Figure A1. Separate models were run for effects on each age group in the studies (children, younger adults, older adults). The models were run using PROCESS in SPSS (Model 4; Hayes, 2013).[Fig fig9]

### Study 1

All β coefficients, including 95% confidence intervals for unstandardized coefficients, are shown in Table A1.[Table tbl8] [Table tbl9]

The analyses showed that for children, the risk message significantly decreased probability perceptions for infection, β = −0.24, *p* = .019, but not for severe symptoms, β = −0.01, *p* = .944, but only probability perceptions for severe symptoms increased advice for children to stay home, β = 0.14, *p* = .014. The mediation effects were not significant, with an overall indirect effect of -0.01, 95% CI [−0.06, 0.03].

For younger adults, the risk message did not affect probability perceptions, β_effect of risk reminder on probability perception of infection_ = −0.13, *p* = .200, β_effect of risk reminder on probability perception of severe symptoms_ = −0.01, *p* = .900, nor the advice to stay home, β = −0.11, *p* = .290. There were no significant mediation effects, with the indirect effects were around 0.01.

For older adults, the risk message did not affect probability perceptions, β_effect of risk reminder on probability perception of infection_ = 0.02, *p* = .876, β_effect of risk reminder on probability perception of severe symptoms_ = −0.001, *p* = .992, nor the advice to stay home, β = 0.01, *p* = .935. Again, mediation effects were superfluous, with all indirect effects close to 0.

### Study 2

The analyses did not find that the risk reminder had a significant effect on probability perceptions for either group (at increased risk, β_effect of risk reminder on probability perception of infection_ = −0.13, *p* = .369, β_effect of risk reminder on probability perception of severe symptoms_ = −0.12, *p* = .386; or not at increased risk, β_effect of risk reminder on probability perception of infection_ = −0.08, *p* = .554, β_effect of risk reminder on probability perception of severe symptoms_ = -0.05, *p* = .727). Only the probability perception for severe symptoms significantly increased advice to both groups to stay home, β_effect of probability perception of severe symptoms, increased risk group_ = 0.18, *p* = .032, β_effect of probability perception of severe symptoms, not increased risk group_ = 0.19, *p* = .013. None of the indirect effects were significant, for children: −0.02 [−0.09, 0.05]; for older adults: −0.01 [−0.06, 0.02]. We therefore found no significant mediation.
