# Supplementary material for: Ambiguity and Unintended Inferences About Risk Messages for COVID-19
Source: J Exp Psychol Appl. 2022 May 19;28(3):486–508. doi: 10.1037/xap0000416 (PMC9899423; doi:10.1037/xap0000416)
Supplement: Supplementary file 1 [file xap0000416_sm.docx]

**Supplementary Material**

We report a supplementary analysis on whether participants in the studies interpreted “some people are at increased risk from coronavirus” in the risk interpretation question as having a greater chance of *carrying* the virus.

Table S1 shows the breakdown of participants who believed the “increased risk” meant carrying the virus, and whether they also believed it meant another interpretation (infection and/or hospitalisation). For all three studies, participants who did believe it meant carrying the virus also believed it meant the other interpretations (and ~90% believed it meant all). No one believed it meant carrying the virus but not the other interpretations, although 1 person in Studies 2 and 4 were uncertain about infection and hospitalisation but thought it meant carrying the virus.

Table S1

Participants’ responses to whether being “at increased risk” from coronavirus meant having a greater chance of carrying the virus.

| *Being “at increased risk” from coronavirus means having a greater chance of…* | | | | |
| --- | --- | --- | --- | --- |
|  | Study 1  (*n* = 396) | Study 2  (*n* = 399) | Study 3  (*n* = 432) | Study 4  (*n* = 474) |
| Carrying the virus  Of which: | 85 | 82 | 102 | 102 |
| *Carrying the virus only* | 0 | 1 | 0 | 1 |
| *Carrying the virus and being infected* | 11 | 4 | 7 | 4 |
| *Carrying the virus and being hospitalisation* | 1 | 4 | 4 | 8 |
| *All three are increased* | 77 | 71 | 91 | 89 |

Table S2 shows the breakdown of participants who believed the “increased risk” meant carrying the virus in relation to whether they also thought it meant being infected with the virus. As shown in Table S2, among participants who held the “being infected” interpretation, around 35-40% also believed it meant carrying the virus as well, whereas among participants who did not hold the “being infected” interpretation, less than 5% of them also held a “carrying the virus” interpretation.

Table S2

Participants’ responses to whether being “at increased risk” from coronavirus meant having a greater chance of carrying the virus if one already believes the risk means a greater chance of being infected.

| *Being “at increased risk” from coronavirus means having a greater chance of …* | | | | |
| --- | --- | --- | --- | --- |
|  | Study 1  (*n* = 396) | Study 2  (*n* = 399) | Study 3  (*n* = 432) | Study 4  (*n* = 474) |
| Being infected with the virus: Yes | 222 | 220 | 260 | 234 |
| *… and carrying the virus* | 84 | 75 | 98 | 93 |
| Being infected with the virus: No/don’t know | 174 | 179 | 169 | 240 |
| *… and carrying the virus* | 1 | 5 | 4 | 9 |

*Note:* Data for this question was missing from three participants in Study 3.

We report exploratory ANOVAs on whether saying “yes” to this interpretation (vs. “no” or “don’t know”) affected risk perceptions of infection to different groups of individuals.

**Study 1**

Participants who believed risk meant carrying the virus perceived higher rates of infection overall than those who did not, *F*(1, 392) = 10.68, *p* = .001, η^2^_P_ = 0.03. The interpretation variable also interacted with group vulnerability, *F*(1, 392) = 20.04, *p* < .001, η^2^_P_ = 0.05. Those who believed it perceived a higher rate of infection to vulnerable individuals than those who did not, whereas risk perceptions for non-vulnerable individuals did not differ across interpretations. There was no significant interaction between interpretations and message condition, *F*(1, 392) = 0.06, *p* = .802, η^2^_P_ < .001, nor a significant three-way interaction, *F*(1, 392) = 0.02, *p* = .886, η^2^_P_ < .001.

**Study 2**

Participants who believed risk meant carrying the virus perceived higher rates of infection overall than those who did not, *F*(1, 346) = 13.41, *p* = .801, η^2^_P_ = 0.04. Interpretations did not interact with any other variable, *p*s for all interactions > .10.

**Study 3**

Participants who believed risk meant carrying the virus perceived higher rates of infection overall than those who did not, *F*(1, 402) = 18.78, *p* < .001, η^2^_P_ = 0.05. The interpretation variable interacted with group vulnerability, *F*(1, 402) = 28.12, *p* < .001, η^2^_P_ = 0.07. Those who believed it perceived a higher rate of infection to vulnerable individuals than those who did not, whereas risk perceptions for non-vulnerable individuals did not differ across interpretations. There was no significant interaction between interpretations and message condition, *F*(1, 402) = 0.07, *p* = .798, η^2^_P_ < .001, nor a significant three-way interaction, *F*(1, 402) = 0.24, *p* = .623, η^2^_P_ = .001.

**Study 4**

The interpretation variable interacted with group vulnerability, *F*(1, 470) = 55.43, *p* < .001, η^2^_P_ = 0.11. Those who believed it perceived a higher rate of infection to vulnerable individuals than those who did not, whereas risk perceptions for non-vulnerable individuals did not differ across interpretations. There was an interaction between interpretation as carrying the virus and message condition, *F*(1, 470) = 6.17, *p* = .013, η^2^_P_ = 0.01. For those who did not interpret the risk as that of carrying the virus, risk perceptions for infection were higher for the improved message than the original message, whereas the message did not have an effect on risk perceptions for those who believed the risk meant carrying the virus. The three-way interaction with group vulnerability was not significant, *F*(1, 470) = 0.20, *p* = .659, η^2^_P_ < .001.
